# Infiltration characteristics and regulatory mechanisms of CD8^+^ T lymphocytes in solid tumors: spatial distribution, biological functions, and interactions with the immune microenvironment

**DOI:** 10.3389/fimmu.2025.1661545

**Published:** 2025-10-01

**Authors:** Peng Ouyang, Jianhong Zhang, Xiao He, Congcong Yang, Dingcheng Zeng, Daofeng Xu

**Affiliations:** ^1^ Department of Hepatobiliary Surgery, First Affiliated Hospital of Gannan Medical University, Ganzhou, China; ^2^ Ganzhou Key Laboratory of Hepatocellular Carcinoma, The First Affiliated Hospital of Gannan Medical University, Ganzhou, China; ^3^ The First Clinical Medical School of Gannan Medical University, Ganzhou, China

**Keywords:** CD8+ T cells, tumor microenvironment, cancer-immunity cycle, T-cell infiltration, immunotherapy resistance

## Abstract

CD8^+^ T lymphocytes are central effectors of anticancer immunity. Their abundance and spatial distribution within solid tumors are strongly correlated with patient prognosis and response to immune-checkpoint inhibitors (ICIs). Tumors have been categorized into “hot,” “excluded,” and “cold” types based on the infiltration patterns of CD8^+^ T cells, which reflect the underlying immune contexture and therapeutic potential. However, many tumors remain resistant to T-cell infiltration, posing a significant barrier to immunotherapy. This review systematically outlines the seven critical steps of the Cancer-Immunity Cycle that govern CD8^+^ T-cell infiltration: antigen release, antigen processing and presentation, T-cell priming, trafficking through the vasculature, tumor infiltration, target recognition, and cytolytic activity. At each step, tumor-intrinsic and microenvironmental barriers—including low tumor mutational burden, defective antigen-presenting machinery, immunosuppressive cytokines (e.g., TGF-β, IL-10), abnormal vasculature, fibroblast-derived extracellular matrix, and inhibitory cell populations (e.g., Tregs, MDSCs, TAMs)—can stall the immune response. We further discuss the roles of immune-checkpoint signaling, metabolic competition, and suppressive cell networks in shaping T-cell exhaustion and exclusion. Cutting-edge technologies—such as single-cell RNA-sequencing, spatial transcriptomics, imaging mass cytometry, and TCR repertoire profiling—have revealed spatial and functional heterogeneity within intratumoral CD8^+^ T cells and informed the design of rational combination therapies. Understanding and targeting these barriers is critical for converting immune-cold tumors into immune-infiltrated, therapy-responsive states. We conclude with a perspective on the future of immunoengineering and immune-atlas integration to optimize CD8^+^ T-cell–based interventions in solid tumors.

## 1 Introduction

**Figure 1:**
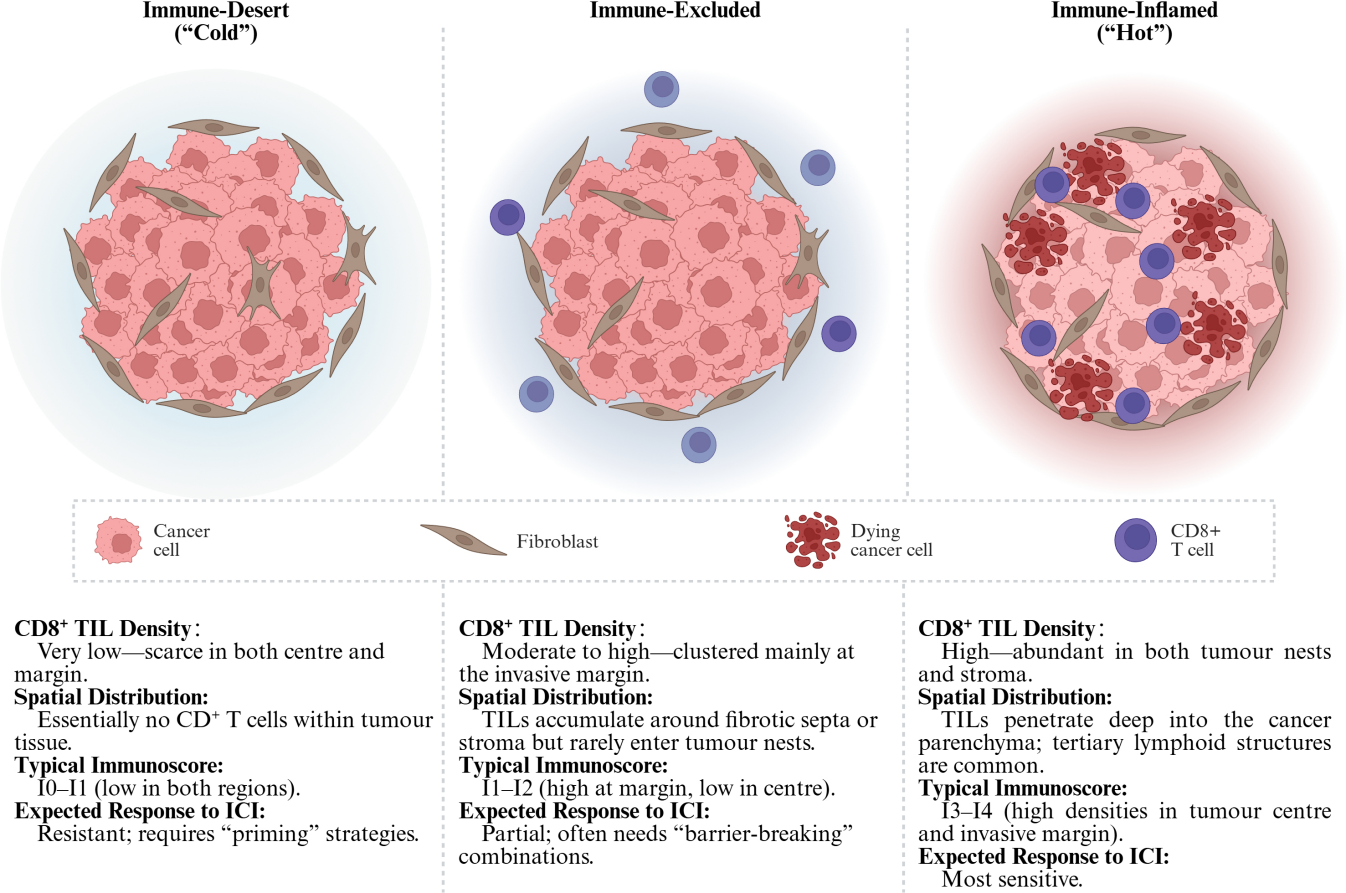
Tumor immune phenotypes. The Immunoscore classifies tumors into five grades (I0–I4) by quantifying the density of CD3^+^/CD8^+^ T cells in both the tumor center (CT) and the invasive margin (IM) (3). Tumors with I3–I4 scores typically correspond to “hot” tumors, while I0–I1 grades are commonly associated with “cold” or “excluded” phenotypes. TIL, Tumor-Infiltrating Lymphocytes; ICI, Immune Checkpoint Inhibitors. (Created in BioRender. Lan, L (2025). https://BioRender.com/ucfl99z).

CD8^+^ T lymphocytes are the chief cytotoxic effectors of antitumor immunity. Numerous cohort studies—including a meta−analysis of 18 700 patients across 17 solid−tumor types—demonstrate that a high density of intratumoral CD8^+^ T cells is one of the strongest predictors of prolonged overall survival and durable responses to immune−checkpoint blockade (1). Conversely, tumors with sparse or peripherally sequestered CD8^+^ T cells (so−called “immune−cold” or “immune−excluded” phenotypes) correlate with poor outcomes and primary resistance to immunotherapy (2). These observations have led to a functional taxonomy of solid tumors as “hot,” “altered–excluded,” or “cold,” largely defined by the quantity and spatial localization of infiltrating CD8^+^ T cells (**Figure 1**) (4–7). Understanding why some tumors remain impermeable to these cells is therefore central to improving current immunotherapies.

This review synthesizes current knowledge on the patterns and regulators of CD8^+^ T−cell infiltration in solid tumors. We will (I) dissect the anatomic and molecular barriers—vascular, stromal, metabolic, and immunologic—that dictate spatial distribution; (II) examine how immune−checkpoint signaling, suppressive myeloid and stromal populations, and nutrient or oxygen deprivation modulate CD8^+^ T−cell fate and function; and (III) highlight emerging multi−omic technologies—such as single−cell RNA sequencing, spatial transcriptomics, high−multiplex imaging mass cytometry, and T−cell−receptor repertoire profiling—that are reshaping our ability to map these processes *in situ* and to translate mechanistic insights into therapeutic strategies capable of converting “immunotherapy-resistant” tumors into “immunotherapy-responsive” ones.

## 2 CD8^+^ T-cell infiltration patterns and spatial landscapes in solid tumors

### 2.1 A Tripartite immune-phenotype continuum — inflamed, excluded, desert

Large-scale profiling of solid tumors has converged on three recurrent patterns of CD8^+^ T-cell infiltration. Immune-inflamed (“hot”) tumors display dense CD8^+^ TIL dispersed throughout the cancer nests and stroma, often accompanied by an interferon-γ–rich cytokine milieu (8–10). Immune-excluded lesions harbor abundant CD8^+^ T cells, but these cells are trapped at the invasive margin or within fibrotic septa and seldom penetrate the parenchyma (11). At the opposite extreme, immune-desert (“cold”) tumors show a near-complete paucity of CD8^+^ T cells in both center and periphery (6). This inflamed–excluded–desert continuum is now widely used to stratify the baseline tumor immune contexture and to anticipate responses to immunotherapy (**Figure 1**).

### 2.2 Quantitative and positional metrics — from simple counts to the Immunoscore

Beyond binary “hot/cold” labels, quantitative cut-offs refine prognostication: > 500–750 CD8^+^ T cells/mm^2^ typically denotes high infiltration, whereas < 100 CD8^+^ T cells/mm^2^ signifies low. Crucially, where the cells reside matters. The Immunoscore algorithm, validated across multiple cohorts, integrates CD3/CD8 densities in the tumor center (TC) and IM to yield five tiers (I0–I4); high scores (I3–I4) correlate with prolonged disease-free survival and superior benefit from immune-checkpoint blockade (12). New data indicate that CD8 density at the IM alone can approximate full Immunoscore performance, simplifying routine pathology pipelines (13).

However, it is important to highlight that there is currently no universally accepted consensus definition for the objective classification of tumor immune phenotypes, and existing categorizations (e.g., “hot”, “cold”, “immune-excluded”) may vary considerably across studies. While the Immunoscore represents a significant advance in quantifying immune infiltration, it is primarily validated in and routinely applied to colorectal cancer, limiting its generalizability to other tumor types. As reviewed by Tiwari et al., definitions of immune phenotypes remain heterogeneous across the literature, underscoring the need for standardized, pan-cancer frameworks for immune contexture classification to guide both research and clinical decision-making (14).

### 2.3 Anatomical niches — invasive front, perivascular hubs and tertiary lymphoid structures

Spatial-omics technologies have revealed that CD8^+^ T cells are not randomly dispersed but concentrate in discrete niches (15). At the invasive front, TIL interdigitate with tumor cells undergoing epithelial–mesenchymal transition, positioning them at a critical bottleneck for metastatic spread (16, 17). Perivascular immune hubs—rich in dendritic cells and chemokines such as CXCL9/10—act as staging grounds where newly recruited CD8^+^ T cells are primed and expanded before entering the tumor core (18). Mature tertiary lymphoid structures (TLSs), identifiable by spatial transcriptomics and high-parameter imaging, harbor germinal-center-like B cells, follicular helper T cells and stem-like CD8 progenitors (19); their presence consistently associates with higher intratumoral CD8^+^ T-cell densities and improved clinical outcome across carcinomas such as nasopharyngeal, ovarian and lung cancers (19, 20).

### 2.4 Clinical ramifications and therapeutic leverage

CD8^+^ T-cell abundance and spatial positioning collectively inform prognosis and guide treatment selection. Inflamed tumors respond best to PD-1/PD-L1 or CTLA-4 blockade (11); excluded tumors often require barrier-modulating combinations (e.g., TGF-β or VEGF inhibitors) to enable parenchymal entry; desert tumors benefit from priming strategies—vaccines, oncolytic viruses, STING agonists—to initiate *de-novo* T-cell recruitment before checkpoint inhibition (6). Thus, precise spatial immunophenotyping not only captures tumor biology but also delineates rational avenues to convert “immunotherapy-resistant” landscapes into “immunotherapy-responsive” ones.

## 3 Tumor-related determinants of CD8^+^ T-cell infiltration

### 3.1 Tumor-antigen release

**Figure 2:**
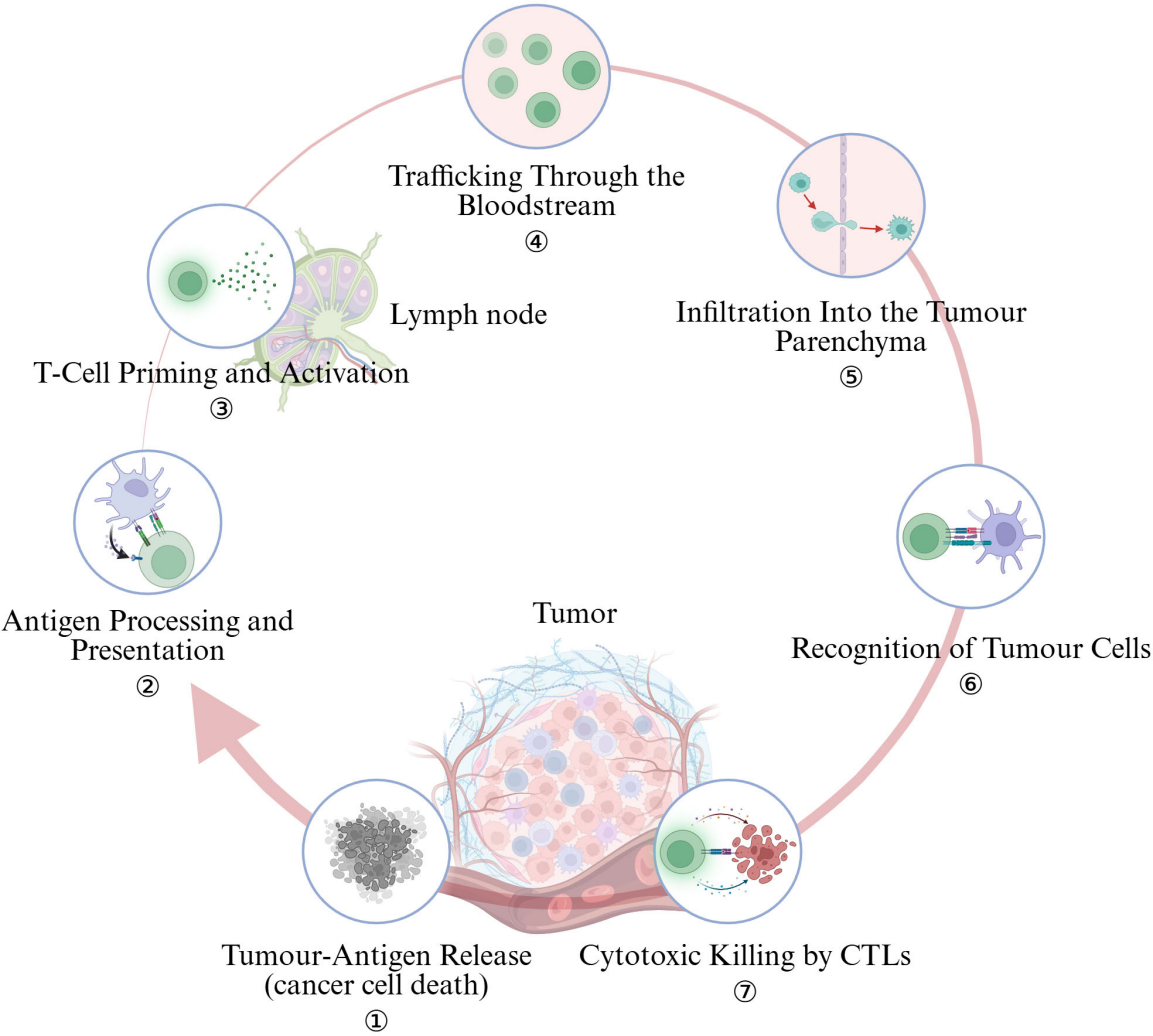
The cancer-immunity cycle encapsulates seven pivotal steps. (Created in BioRender. Lan, L (2025). https://BioRender.com/2294g5o).

Cell-intrinsic mutations, viral integrations or aberrant splicing generate neoantigens that are liberated when tumor cells undergo immunogenic cell death (ICD) triggered by radiotherapy, oxaliplatin, oncolytic viruses or STING/TLR agonists (**Figure 2**). Cancers with few non-synonymous mutations release scant antigenic material, providing little substrate for downstream immune activation. Large cohort analyses show that a high tumor-mutational burden (TMB) correlates with elevated neoantigen load, dense CD8^+^ T-cell infiltration and superior response to PD-1 blockade in lung cancer and other entities (21, 22). Nevertheless, antigen quantity is not the sole bottleneck: melanoma datasets reveal that “cold” and “hot” tumors can express comparable levels of putative antigens, pointing to additional barriers that arrest the cycle (23).

### 3.2 Antigen processing and presentation

After uptake, professional antigen-presenting cells (APCs) must cross-present peptides on HLA-I to prime naïve CD8^+^ T cells. Tumor cells frequently thwart this step by β_2_-microglobulin/TAP loss, HLA-I locus deletions or epigenetic silencing, while VEGF and TGF-β secreted into the micro-environment impair dendritic-cell maturation (23). Restoring type-I/II interferon signaling or pharmacologically demethylating HLA promoters can up-regulate the antigen-presentation machinery and re-ignite the cycle.

### 3.3 T-cell priming and activation

Effective priming in tumor-draining lymph nodes requires Batf3^+^ cDC1s that deliver TCR, co-stimulatory and cytokine “signals 1–3.” Tumors limit this by excluding cDC1s, expanding FOXP3^+^ Tregs that consume CD80/86, and inducing tolerogenic IDO and IL-10 pathways. Complement-component dysregulation or loss of CD40L signaling similarly cripples priming (24). CTLA-4 blockade, FLT3L + poly-ICLC to expand cDC1s, and IDO inhibitors are under clinical exploration to repair this defect.

### 3.4 Trafficking through the bloodstream

Activated T cells egress and follow CXCR3/CCR5 gradients (CXCL9/10, CCL5) to the tumor (25). VEGF-driven, chaotic vasculature down-regulates ICAM-1/VCAM-1 and induces “endothelial anergy,” (26, 27) whereas systemic TGF-β suppresses CXCR3 on effector T cells (28). Anti-angiogenic therapy transiently normalizes vessels and restores adhesion-molecule expression, thereby enhancing T-cell recruitment (29, 30).

Recruitment of cytotoxic T cells relies on chemokine gradients dominated by CXCL9, CXCL10 and CCL5 that engage CXCR3 or CCR5 on circulating CD8^+^ T cells (11). “Hot” tumors typically maintain high interferon-γ signaling and a robust CXCL9/10 axis, whereas “cold” tumors frequently lack these chemo-attractants or preferentially secrete chemokines (e.g., CCL17, CXCL12) that draw suppressive myeloid cells and Treg cells instead (11). Pre-clinical work shows that enforced expression of CXCL9/10, STING agonism or oncolytic viruses can re-establish productive gradients and convert immune-desert lesions into inflamed ones (31).

### 3.5 Infiltration into the tumor parenchyma

Crossing the endothelial barrier is only half the journey; dense, CAF-produced extracellular matrix (ECM) and CXCL12/TGF-β chemokine sinks trap T cells at the invasive margin. CAF-rich desmoplastic cancers (e.g., pancreas) exemplify “immune-excluded” lesions in which CD8^+^ T cells rarely enter the core (32, 33). Strategies such as FAP-targeted depletion, PEGylated hyaluronidase or low-dose radiotherapy that remodel ECM can open stromal corridors for lymphocyte entry.

Most solid tumors develop a highly disorganized vascular network driven by VEGF and other pro-angiogenic cues (34). Tortuous, poorly pericyte-covered vessels express low levels of adhesion molecules (ICAM-1, VCAM-1) and generate erratic blood flow, thereby limiting CD8^+^ T-cell arrest and transendothelial migration. Endothelial cells can also up-regulate Fas ligand or PD-L1, actively deleting or silencing incoming effector T cells. Vascular “normalization” with anti-VEGF or angiopoietin-2 blockade restores vessel integrity and markedly boosts intratumoural T-cell entry in pre-clinical and early clinical studies (34). Beyond the endothelium, cancer-associated fibroblasts (CAFs) deposit dense, cross-linked extracellular matrix that forms a physical “mesh”, sequestering T cells at the invasive front (35); CAF-rich, desmoplastic tumors such as pancreas or cholangiocarcinoma are quintessential immune-excluded lesions (36). Targeting CAFs themselves (for example, FAP-directed approaches) or enzymes that remodel collagen can soften this barrier and facilitate T-cell penetration (37).

### 3.6 Recognition of tumor cells

Effector TILs must engage peptide–HLA-I complexes on target cells. Somatic loss of HLA-I, JAK1/2–IFNGR mutations and tumor-intrinsic WNT/β-catenin signaling all diminish antigen visibility or chemokine output (38, 39). Aberrant WNT/β-catenin suppresses CCL4 and blocks dendritic-cell recruitment; PTEN loss, MYC amplification and stem-like transcriptional programs likewise correlate with immune deserts. Successful immunotherapy may therefore require concurrent targeting of these oncogenic drivers to dismantle “do-not-enter” cues encoded by cancer cells.

### 3.7 Cytotoxic killing by CTLs

Tumor cells and stromal elements secrete a spectrum of factors that dampen T-cell trafficking (40). TGF-β is a central gatekeeper: it stiffens the ECM, suppresses endothelial adhesion molecules and directly curtails T-cell motility; dual blockade of TGF-β and PD-1/PD-L1 can shift immune-excluded tumors toward an inflamed phenotype in mouse and human studies. Additional metabolites—adenosine (via CD39/CD73), kynurenines produced by indoleamine-2,3-dioxygenase (IDO), lactic acid and high extracellular potassium—create a hostile biochemical milieu that impairs T-cell viability and chemotaxis (41). Neutralizing these pathways (e.g., A2A receptor antagonists, IDO inhibitors) is under active clinical evaluation to enhance CD8^+^ T-cell ingress.

Tumor-secreted TGF-β stiffens the ECM, down-regulates endothelial adhesion molecules and directly hampers T-cell motility; dual PD-1/TGF-β blockade can shift immune-excluded tumors toward an inflamed phenotype. Adenosine (CD39/CD73), IDO-derived kynurenines, lactate and high extracellular K^+^ further create a biochemical quagmire that blunts CTL chemotaxis and survival (42, 43). A2A-receptor antagonists and IDO inhibitors are under clinical evaluation to reopen these metabolic choke points.

## 4 Immune-regulatory mechanisms within the tumor micro-environment shaping CD8^+^ T-cell fate

### 4.1 Immune-checkpoint signaling and exhaustion

Continuous antigen exposure, combined with inhibitory receptor engagement, drives CD8^+^ T cells toward a hypofunctional “exhausted” state characterized by diminished cytotoxicity and high expression of PD-1, CTLA-4, TIM-3, LAG-3 and TIGIT (44, 45). Tumor cells, tumor-associated macrophages (TAMs) and endothelial cells up-regulate ligands such as PD-L1 or B7-family molecules, directly silencing T-cell receptor signaling (46). Dual blockade of PD-L1 and TGF-β has shown that relieving co-inhibition and simultaneously dismantling stromal barriers allows expansion of stem-like CD8^+^ precursors and their intratumoural accumulation, converting excluded lesions into responsive, inflamed tumors (47, 48).

### 4.2 Metabolic constraints and biochemical suppressors

Solid tumors reshape nutrient supply and waste removal, creating an extracellular milieu with depleted glucose/amino acids/oxygen and elevated metabolic by-products (e.g., lactate, adenosine, K^+^) that collectively blunt CD8^+^ T-cell proliferation, cytokine production and survival. Quantitative metabolomics of tumor interstitial fluid (TIF) confirms altered metabolite concentrations *in situ*, providing concentration ranges that map onto T-cell dysfunction (49).

#### 4.2.1 Lactate/lactic acidosis

Tumor LDHA-driven glycolysis and monocarboxylate transporter (MCT) export raise extracellular lactate and H^+^. Pathophysiological lactic acidosis directly inhibits CTL and NK function, curtailing cytokines and cytolysis while suppressing TCR-proximal signaling programs including NFAT, with consequent loss of IFN-γ. It also impairs T-cell motility and chemotaxis, reinforcing exclusion. Targeting lactate production/transport (LDHA or MCTs) therefore represents a tractable lever to restore CD8^+^ effector programs (50, 51).

#### 4.2.2 Adenosine: A_2_A-cAMP/PKA and ENT1–pyrimidine axes

Hypoxia and CD39/CD73 activity elevate extracellular adenosine, which engages A_2_A receptors on T cells to raise cAMP–PKA signaling and antagonize TCR pathways (including NFAT), broadly suppressing CTL killing and cytokine output. Clinically, A_2_A antagonists (e.g., ciforadenant) are being tested alone or with PD-(L)1 blockade. In parallel, a recent study shows intracellular adenosine uptake via ENT1 depletes pyrimidines and throttles biosynthesis in activated T cells; pharmacologic ENT1 inhibition restores nucleotide pools and rescues anti-tumor activity—highlighting two complementary adenosine checkpoints (52–55).

#### 4.2.3 Extracellular potassium (the “ionic checkpoint”)

Necrosis-rich tumor cores release K^+^, elevating interstitial [K^+^] and forcing T cells into a nutrient-hoarding, hypoactive state. Mechanistically, high [K^+^]e suppresses TCR-driven Akt–mTOR phosphorylation (via PP2A), limiting effector programs; relieving the K^+^ brake restores CD8^+^ function (56).

#### 4.2.4 Hypoxia and tryptophan–kynurenine (IDO/TDO–AhR) signaling

Hypoxia reshapes T-cell metabolism and fate, contributing to exhaustion features and altering effector capacity; HIF-1α activity tunes CD8^+^ responses in tumors while also promoting immunosuppressive circuits. In parallel, IDO/TDO-driven kynurenine engages AhR to dampen T-cell immunity; blocking this circuit restores responsiveness and synergizes with checkpoint blockade in models (57, 58).

#### 4.2.5 Translational levers (metabolic immuno-engineering)

Strategies under active preclinical/clinical evaluation include: (i) inhibiting lactate production/transport to reduce lactic acidosis; (ii) A_2_A receptor antagonists and ENT1 inhibitors to revoke adenosine-mediated suppression; (iii) approaches that buffer or bypass the K^+^ ionic checkpoint; and (iv) IDO/AhR pathway inhibitors—often combined with PD-(L)1—to reprogram the metabolic contexture and increase intratumoural CD8^+^ T-cell quantity and quality (54, 59).

### 4.3 Soluble cytokines and growth factors

TGF-β is a master suppressor that stiffens the extracellular matrix, down-regulates endothelial adhesion molecules and imprints an exhaustion-prone transcriptional program on CD8^+^ T cells (48, 60); high TGF-β signatures typify immune-excluded tumors and predict poor response to monotherapy ICI (61, 62). VEGF not only fuels aberrant angiogenesis but also induces endothelial “anergy,” further impeding T-cell extravasation (26, 63). Together with IL-10 and prostaglandin-E2, these soluble factors create an anti-inflammatory milieu that limits effector-cell recruitment and survival (64). Combination regimens that co-target VEGF or TGF-β with PD-1/PD-L1 blockade are now actively pursued to dismantle these layered defenses (65, 66).

### 4.4 Immunosuppressive cellular networks

Regulatory T cells (Tregs), myeloid-derived suppressor cells (MDSCs) and TAMs form a cellular triad that orchestrates resistance to CD8^+^ immunity. Tregs accumulate in hypoxic, adenosine-rich niches and secrete IL-10, TGF-β and granzyme-B to dampen local cytotoxic responses; a high Treg/CD8 ratio correlates with shortened survival across many carcinomas (67). MDSCs deplete arginine and cystine, generate nitric oxide and reactive oxygen species, and express checkpoint ligands, thereby stalling CD8^+^ activation and clonal expansion (68). TAMs of an M2-like phenotype produce IL-10 and PD-L1, phagocytose effector cells via FcγR engagement and remodel stroma to favor immune exclusion (69). Therapeutic depletion or functional reprogramming of these suppressor populations — for example, CCR2/CSF1R blockade for TAMs or arginase/iNOS inhibition for MDSCs — synergizes with checkpoint inhibitors by unleashing CD8^+^ TILs (70, 71).

### 4.5 Integrated impact on therapy

These overlapping inhibitory layers—checkpoint engagement, metabolic starvation, soluble suppressors and suppressive cell populations—conspire to limit both the quantity and quality of intratumoural CD8^+^ T cells. Rational combination approaches that simultaneously relieve co-inhibition, rectify nutrient stress, neutralize soluble cytokines and curtail suppressor cells are therefore essential to transform immune-”immunotherapy-resistant” tumors into T-cell-inflamed, therapy-responsive states.

## 5 Biological functions of CD8^+^ T cells and their antitumor actions

### 5.1 Cytotoxic armamentarium—precision killing of malignant cells

Upon T-cell receptor recognition of peptide–MHC-I complexes on tumor cells, activated CD8^+^ T cells release perforin to create transient pores and deliver granzyme B and related serine proteases that trigger caspase-dependent apoptosis (72). Parallel death-receptor pathways—Fas (FasL) and TNF-related apoptosis-inducing ligand—provide redundancy, ensuring cytolysis even when one route is impaired (73). High intratumoural expression of perforin–granzyme transcripts correlates with better survival across carcinomas and can be boosted pharmacologically (e.g., mTOR modulation) to accelerate target-cell elimination (74).

### 5.2 Cytokine orchestration—remodeling the tumor micro-environment

Beyond direct lysis, effector CD8^+^ T cells act as “mobile cytokine factories.” Interferon-γ (IFN-γ) up-regulates tumor MHC-I and components of the antigen-processing machinery (75), amplifies CXCL9/10 chemokine gradients that recruit additional CXCR3^+^ T cells, and exerts anti-angiogenic and anti-proliferative effects on neoplastic and stromal cells (76). Tumor-wide, bystander IFN-γ signaling can propagate hundreds of microns, converting immunologically “cold” niches into inflamed ones (77, 78). Conversely, sustained STAT1/IRF1 activation may drive adaptive resistance (e.g., PD-L1 up-regulation), highlighting the need for balanced cytokine tone (76).

### 5.3 Functional states—effector, resident-memory, and stem-like exhausted subsets

Intratumoural CD8^+^ T cells are heterogeneous. Classical short-lived effector cells provide immediate cytotoxicity but decline rapidly. Tissue-resident memory (T_RM_) cells, distinguished by CD69 and CD103, lodge long-term within epithelial niches, secrete IFN-γ “on site,” and independently predict favorable prognosis in multiple solid tumors (79–81). In chronically antigenic TMEs, a hierarchical exhaustion program emerges: a TCF1^high^ stem-like progenitor pool (TPEX) self-renews and seeds terminally exhausted PD-1^hi^ TIM-3^+^ cells that retain limited killing capacity (82). Checkpoint blockade preferentially expands TPEX cells, explaining why their baseline frequency foreshadows clinical benefit (83).

### 5.4 Positive-feedback loops—amplifying immunity and provoking epitope spread

Tumour-cell death initiated by CD8^+^ T cells releases danger-associated molecular patterns (DAMPs) and neoantigens that fuel dendritic-cell activation and cross-presentation, a process termed epitope spreading (84). IFN-γ further licenses intratumoural dendritic cells, while TNF-α and GM-CSF reshape myeloid composition toward an M1-like phenotype. These events create a feed-forward circuit that recruits fresh waves of cytotoxic T cells, broadens antigenic breadth, and can ultimately overcome tumor heterogeneity. Therapeutic strategies that sustain this circuit—such as local cytokine delivery, STING agonists, or agents that preserve TCF1^+^ precursors—are under active clinical exploration (85).

Collectively, CD8^+^ T cells exert multifaceted antitumor effects—direct lysis, cytokine-mediated remodeling, and adaptive amplification—whose efficacy depends on maintaining a balanced repertoire of effector, memory, and stem-like subsets within a permissive TME.

## 6 Cutting-edge technologies empowering CD8^+^ T-cell infiltration research

### 6.1 Single-cell transcriptomics unlocks TIL heterogeneity 

**Figure 3:**
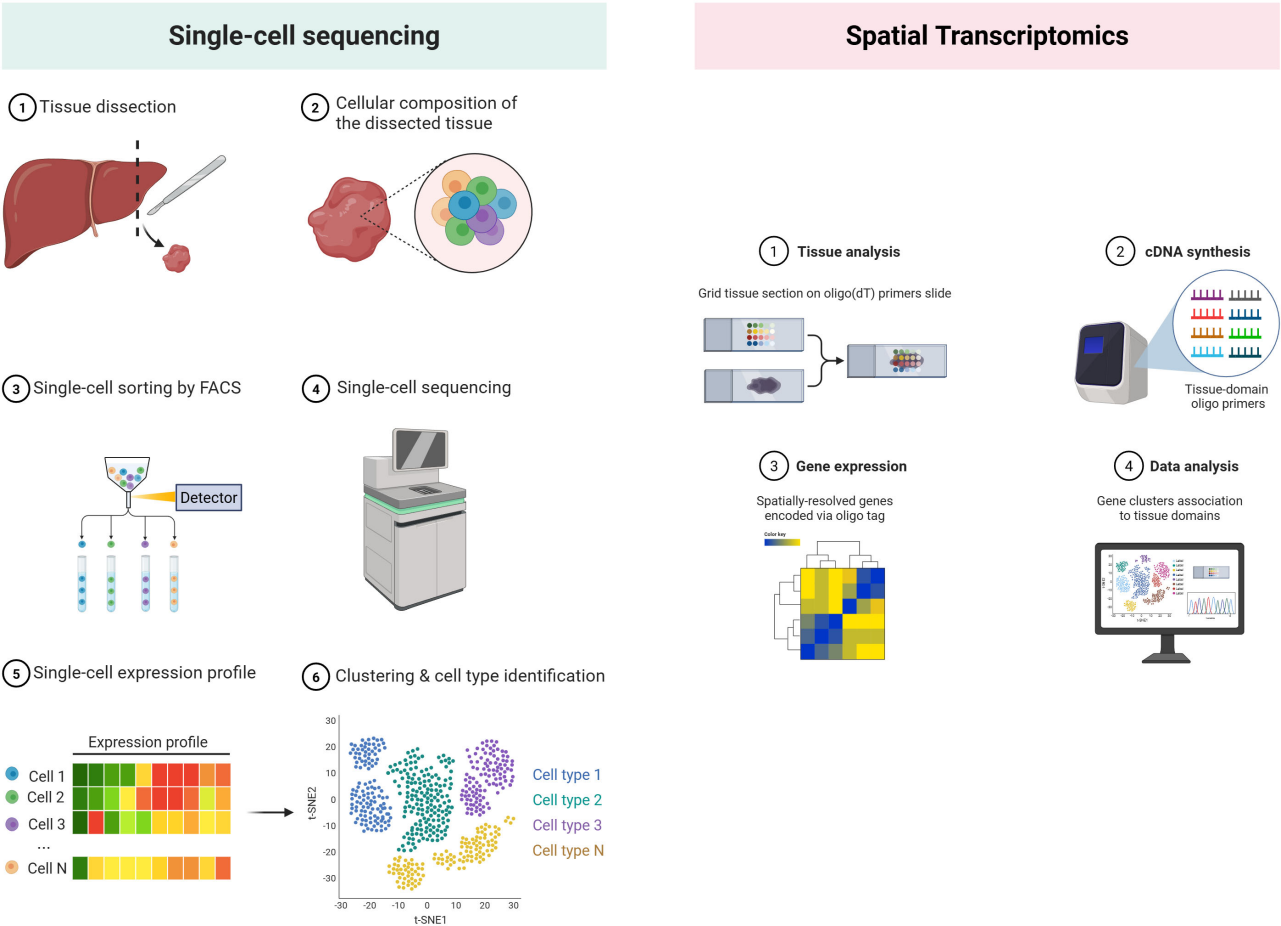
Single-cell sequencing vs spatial transcriptomics. (Created in BioRender. Lan, L (2025). https://BioRender.com/2294g5o).

Single-cell RNA-sequencing (scRNA-seq) has re-defined intratumoural CD8^+^ T-cell taxonomies, resolving short-lived effectors, tissue-resident memory (T_RM_), and the TCF1^+^ stem-like exhausted (T_PEX_) progenitors that seed durable responses to immunotherapy (81). Historical development. The first proof-of-concept scRNA-seq was reported by Tang et al. in 2009, analyzing mouse blastomeres with manual micromanipulation (86). SMART-Seq/Smart-Seq2 subsequently improved full-length transcript coverage and sensitivity (87, 88). A transformative leap came in 2015 with droplet microfluidic platforms—Drop-seq and inDrop—which enabled barcoding of thousands of cells in nanoliter droplets (89, 90). Commercial Chromium technology (10x Genomics) further standardized droplet scRNA-seq and introduced unique molecular identifiers (UMIs) for digital transcript counting (91). These advances dropped per-cell costs by >100-fold and unlocked routine immune-profiling of complex tissues (**Figure 3**).

Recent pan-cancer atlases and disease-focused studies—e.g., in pediatric glioma—demonstrate how shifts in these subsets track with clinical outcome and immune-checkpoint blockade efficacy (92). Integrated scRNA/TCR modalities further couple transcriptional states to clonal evolution, pinpointing which clones breach immune exclusion versus those stalled at the invasive margin.

### 6.2 Spatial transcriptomics maps chemokine landscapes and physical barriers 

Early attempts to retain positional gene information relied on laser-capture microdissection coupled with microarrays, limiting throughput and resolution. A conceptual breakthrough came in 2016, when Ståhl et al. arrayed spatially bar-coded oligo-dT spots on glass slides, enabling transcript capture directly from intact tissue sections and co-registration with histology (93). The advent of droplet printing and bead-based strategies soon pushed resolution from 100 µm spots to near-single-cell scales: Slide-seq (94) placed 10-µm DNA-bar-coded beads onto adhesive slides. Commercial Visium (10x Genomics) standardized 55-µm bar-coded spots and paired them with user-friendly software, catalyzing widespread adoption across oncology and immunology. Parallel imaging-based platforms—seqFISH+, MERFISH and Xenium—achieved sub-micron resolution by cyclic *in-situ* hybridization, but at the cost of limited gene panels, making array-based methods preferable for unbiased chemokine mapping (95, 96) (**Figure 3**).

Next-generation spatial transcriptomics (ST) overlays gene expression on intact tumor sections at near-single-cell resolution. Analytic frameworks such as ReMiTT trace T-cell migratory paths and identify chemokine “highways” (CXCL9/10, CCL5) as well as stromal “cul-de-sacs” enriched for TGF-β or NOS2/COX2 that impede penetration (97). Combining ST with histology or 3D multiscale modelling charted how TLS-rich niches nucleate CD8^+^ T-cell clusters and forecast prolonged survival across carcinomas (98).

### 6.3 Multiplexed imaging visualizes cellular choreography *in situ*

Imaging mass cytometry (IMC) and CODEX antibody cycling now quantify 40–60 proteins per 1-μm pixel, preserving spatial context (99–102). These platforms reveal perivascular “immune hubs” where dendritic cells license newly arrived CD8^+^ T cells, as well as CAF-lined stromal corridors that fence them out. In breast, lung and colorectal cancers, IMC-derived interaction maps of CD8^+^ T cells with NOS2^+^/COX2^+^ tumor islands or PD-L1^+^ macrophage cords stratify responders to PD-1 therapy. Such high-parameter imaging feeds directly into computational tissue atlases that nominate barrier-breaking or TLS-inducing combination regimens.

### 6.4 T-cell-receptor sequencing traces clonal dynamics 

Early repertoire studies used CDR3‐length “spectratyping,” capturing only a crude size distribution. The first high-throughput TCRβ deep-sequencing was reported by Robins et al. in 2009, using multiplex PCR and 454 pyrosequencing to enumerate >200–000 clonotypes in leukemia patients (103). ImmunoSEQ (Adaptive Biotechnologies, 2011) then standardized bulk-repertoire profiling across thousands of samples. A key leap to single-cell resolution came in 2014 when Stubbington et al. paired SMART-Seq cDNA with nested PCR to recover full α/β chains from individual T cells (104). Droplet microfluidics soon enabled scalable capture: 10x Genomics Chromium V(D)J (2017) bar-codes transcripts and links paired TCRs to whole-transcriptome profiles (91). Recent innovations—VIDJIL-airr, TraCeR2 and spatial bar-coding (Slide-TCR-seq, 2022)—now assign clonotypes to precise tissue coordinates, closing the gap between repertoire and geography.

Bulk and single-cell TCR-seq profile the breadth, depth and spatial provenance of tumor-specific clones. Diverse, expanded TCR repertoires associate with inflamed phenotypes, whereas oligoclonal or non-overlapping repertoires typify deserts (105). Longitudinal TCR tracking exposes clonal replacement after checkpoint blockade and reveals whether new infiltrates originate from peripheral reservoirs or *in situ* expansion (106, 107). When stitched to ST or IMC, clonotype barcodes register where hot-spot clones accumulate and which stromal routes they exploit or avoid.

### 6.5 Epigenomic and multi-omic integration defines developmental bottlenecks 

Single-cell ATAC-seq and joint RNA/ATAC/TCR platforms chart chromatin accessibility underlying exhaustion trajectories; for example, CXCR4 or Id2 disruption reshapes the epigenetic landscape, delaying T_PEX_→terminal transition and enhancing infiltration (108). Machine-learning pipelines that fuse scRNA, ST, multiplex imaging and ATAC layers now predict rate-limiting ligands, metabolic sinks and physical barriers, guiding rational multiplexed interventions (99).

Collectively, these synergistic technologies transform static “snapshot” views into dynamic, multi-scale maps of how CD8^+^ T cells navigate, persist and function within solid tumors—informing precision strategies to ignite, channel and sustain antitumor immunity.

## 7 Clinical correlates and immunotherapy resistance: from bench to bedside

Real-world clinical experience with immune-checkpoint inhibitors highlights the importance of addressing immunotherapy resistance, which can be broadly classified into primary resistance (no initial clinical response) and secondary/acquired resistance (progression after initial benefit). Primary resistance is frequently associated with non-inflamed or immune-excluded tumors characterized by low TMB, defective antigen presentation (e.g., β2M mutations, MHC class I loss), and immunosuppressive cytokines such as TGF-β and IL-10, leading to ineffective T-cell priming and infiltration (109, 110). Secondary resistance can develop through tumor immune editing, neoantigen loss, interferon-γ signaling pathway mutations, upregulation of alternative inhibitory checkpoints (TIM-3, LAG-3, TIGIT), or recruitment of immunosuppressive myeloid cells (111, 112). Strategies under clinical investigation to overcome these barriers include dual checkpoint blockade (anti-PD-1 plus anti-CTLA-4 or anti-LAG-3), VEGF/angiogenesis inhibitors to normalize tumor vasculature, STING agonists and oncolytic viruses to induce *in situ* immunogenic cell death, and adoptive cell therapy such as TILs or engineered TCR-T cells, with several trials demonstrating improved response rates in immunotherapy-resistant tumors (113, 114). Nonetheless, major challenges remain, including the lack of consensus immune phenotype classification across tumor types, limited availability of robust predictive biomarkers, immune-related adverse event management in combination regimens, and the need for adaptive trial designs integrating real-time biomarker monitoring (7). Addressing these issues will be key to translating novel immune-engineering approaches and spatial immune atlas data into durable clinical benefit, ultimately achieving the conversion of immunotherapy-resistant tumors into therapy-responsive states.

## 8 Future perspectives

Over the next decade, deciphering and therapeutically exploiting the spatial and functional heterogeneity of CD8^+^ T cells will require four converging lines of progress—now augmented by an AI-first bioinformatics layer.

### 8.1 Multi-scale data integration—now AI-enabled

High-resolution single-cell, spatial-omics, proteomic, metabolomic and epigenomic platforms are mature enough to be harmonized through machine-learning pipelines. Emerging spatially aware AI tools—DeepST, CellCharter, and related graph/latent-variable models—integrate single-cell states with tissue context to infer cell–cell communication, niche topology and migratory routes. When coupled to longitudinal sampling, these models can assemble predictive immune atlases that capture the dynamic interplay among T-cell clones, stromal niches and metabolic landscapes across treatment time-points. Such atlases should inform in silico trials, accelerate hypothesis testing, and refine patient-selection algorithms for combination therapy.

### 8.2 Mechanism-guided therapeutic engineering

Rational “three-layer” regimens are emerging: (i) priming agents that ignite *de novo* T-cell recruitment in immune-desert tumors (e.g., RNA vaccines, STING or TLR agonists); (ii) barrier-modulating drugs that normalize vasculature or remodel CAF-derived matrix to convert immune-excluded lesions into inflamed ones; and (iii) maintenance strategies—checkpoint blockade, metabolic rewiring, or IL-2/IL-7/IL-15 variants—that sustain stem-like precursors and prevent terminal exhaustion. Synthetic-biology approaches such as logic-gated CAR-T cells, mRNA-encoded cytokine factories and conditionally active bispecific antibodies promise unprecedented spatial and temporal control of effector function while minimizing on-target/off-tumor toxicity.

### 8.3 Real-time biomarkers and digital twins

Non-invasive biomarkers (circulating TCR clonotypes, cell-free RNA/DNA, metabolic tracers) should be linked to AI-derived spatial features to track infiltration kinetics and functional states during therapy. Iterating these signals into patient-specific digital twins may enable adaptive dosing and early switching between priming, barrier-modulating, and maintenance layers.

### 8.4 Translational and regulatory considerations for clinical implementation

To move AI-enabled spatial immunology into the clinic, several hurdles must be addressed:(a) Model robustness & generalizability: pre-specify training/validation datasets, perform cross-site testing, and quantify batch effects across platforms and staining protocols; (b) Interpretability & actionability: provide saliency on which spatial features (e.g., perivascular hubs, TLS density, CAF corridors) drive predictions, and map them to trial-eligible interventions; (c) Data governance & privacy: adopt harmonized ontologies and secure data standards; consider federated or privacy-preserving learning for multi-center studies; (d) Regulatory pathway: define software-as-a-medical-device requirements, version control, drift monitoring, and prospective performance benchmarks aligned with clinical endpoints; (e) Reproducible pipelines: containerize end-to-end workflows from raw images/sequencing to clinical reports; release validation kits for external laboratories.

By fusing AI-driven multimodal integration with mechanism-guided therapeutic engineering—and by building a clear regulatory path—the field can transform immune-cold tumors into immune-hot, treatment-sensitive diseases. Success will hinge on interoperable data, interpretable models, and prospective trials that treat computational predictions as testable, patient-benefitting hypotheses.

## 9 Concluding remarks

CD8^+^ T cells lie at the heart of effective anticancer immunity, yet their access to—and performance within—solid tumors are governed by a complex network of vascular, stromal, metabolic and immunological barriers. Recent technological advances have illuminated previously hidden layers of regulation, revealing discrete spatial niches, lineage hierarchies and adaptive feedback loops that dictate therapeutic responsiveness. By integrating these insights with mechanism-guided interventions, the oncology community now has a feasible roadmap to transform immune-cold tumors into immune-hot, treatment-sensitive diseases. Success will hinge on interdisciplinary collaboration, systematic data sharing and careful clinical translation, but the goal is clear: to harness the full cytotoxic and immunomodulatory potential of CD8^+^ T cells for durable cancer control and, ultimately, cure.
